# ZnCl_2_ Incorporated into Experimental Adhesives: Selected Physicochemical Properties and Resin-Dentin Bonding Stability

**DOI:** 10.1155/2017/5940479

**Published:** 2017-11-15

**Authors:** Giselle Soares Almeida, Eduardo Moreira da Silva, José Guilherme Antunes Guimarães, Rayssa Nogueira Lamego da Silva, Glauco Botelho dos Santos, Laiza Tatiana Poskus

**Affiliations:** Analytical Laboratory of Restorative Biomaterials (LABiom-R), School of Dentistry, Universidade Federal Fluminense, Niterói, RJ, Brazil

## Abstract

The aim of this study was to evaluate the degree of conversion (DC%), water sorption (WS), solubility (SO), and resin-dentin bonding stability of experimental adhesive systems containing ZnCl_2_. Different concentrations (wt.%) of ZnCl_2_ were added to a model etch-and-rinse adhesive system consisting of BISGMA, HEMA, UDMA, GDMA, water, and ethanol: Zn0 (0%-control group); Zn2 (2%); Zn3.5 (3.5%); and Zn5 (5%). Adper Single Bond 2 (SB) was used as commercial reference. The samples were light cured for 20s using a quartz-tungsten-halogen unit (650 mW/cm^2^). DC% (*n* = 5) was measured using FT-IR spectroscopy, and WS and SO (*n* = 5) were calculated based on ISO4049. Microtensile bond strength (*μ*TBS) and nanoleakage (NL) were measured after 24 h and 12 months of water storage (*n* = 10). Data were analyzed using ANOVA and Tukey's HSD test (5%). Zn5 presented the lowest DC% and the highest WS and SO (*p* < 0.05). Zn0 and Zn2 presented statistically similar DC%, WS, SO, and immediate *μ*TBS. All adhesives containing ZnCl_2_ maintained a *μ*TBS stability after 12 months, but only Zn2 and Zn3.5 did not suffer an increase in NL. SB presented the highest immediate *μ*TBS but the greatest reduction after 12 months (*p* < 0.05). The addition of 2 wt.% of ZnCl_2_ in adhesive formulations seems to be a promising way to improve the resin-dentin bonding stability. Higher concentrations than 2 wt.% could impair some physicochemical properties.

## 1. Introduction

The stability of the dentin-adhesive interface is one of the main factors responsible for the longevity of the resin composite restorations of teeth. However, despite the exponential improvements introduced to the recent adhesive systems [1, 2], the maintenance of the integrity of the dentin-adhesive interfaces still remains as a challenge in the clinical practice [3, 4]. The degradation of the dentin-adhesive interfaces can happen through two paths. In one path, the polymer adhesive structures formed by the monomers present in the adhesive formulation can suffer a hydrolytic breakdown [5, 6]. A second path involves the degradation of the exposed collagen fibrils, due to poor infiltration of the adhesive monomers within the demineralized dentin [6–8]. This last one has been proposed as one of the major reasons for the long-term degradation of the hybrid layer and the consequent drop in the resin-dentin bonding [9, 10].

The degradation of the collagen fibrils has been related to the enzymatic attack by the matrix metalloproteinases (MMPs) present in dentine and saliva [4, 8, 11]. MMPs are constituted of a group of host-derived enzymes (endopeptidases) dependent on Ca^++^ and Zn^2+^ to maintain their tertiary structure and activation sites, being capable of degrading the components of the extracellular matrix and basal membranes. During the dentinogenesis, MMPs are synthesized and remain entrapped in a latent state within the matrix of mineralized dentin [12, 13]. However, clinically they can be activated by the partial dentin demineralization by acids, thereby starting the mechanism of degradation of exposed collagen fibril network [13–16].

Based on these deleterious effects, strategies such as the use of synthetic MMP-inhibitors as an additional step for dentin treatment or added to commercially available adhesive systems have been proposed to postpone the action of MMPs on the exposed collagen fibrils [17–19]. More recently, the use of adhesives containing Zn to modulate the collagen fibrils degradation by MMPs has also shown encouraging results [20–25]. The collagen structure has four binding sites for Zn at the same location as the cleavage sites of collagenases [26]. Thus, Zn may act as a competitive inhibitor for the MMPs as they can bind to these MMP cleavage sites [27]. Besides this MMP inhibition effect, previous studies have shown that Zn also presents a metabolic effect on the mechanism of hard tissue mineralization that can induce the biomineralization of the collagen fibrils unprotected by the adhesive monomers [20, 25]. Both mechanisms can contribute to the stability of the dentin-adhesive polymer interface.

The previous studies that have analyzed the effect of Zn salts on dentin bonding have added ZnO [21, 23–25], ZnN_3_ [22], Zn-methacrylate [21], and ZnCl_2_ [23, 24, 28], inside commercially available [22–25] or experimental adhesive systems [21]. Among these, ZnCl_2_ is the one that presents a very fast rate of dissolution [20] which may result in a higher saturation of Zn^2+^ into the adhesive system. Moreover, irrespective of these former studies, there is no published data about the ideal concentration of this salt regarding improvements to dentin bonding. Therefore, the purpose of this study was to formulate experimental adhesive systems containing different concentrations of ZnCl_2_, to evaluate their resin-dentin bonding stability and nanoleakage and to characterize some physicochemical properties (DC%, WS, and SO). The experimental hypotheses tested were the following: (1) the DC%, WS, and SO are affected by the adhesives containing different concentrations of ZnCl_2_ and (2) the resin-dentin bonding stability and nanoleakage over a period of 1 year are affected by adhesives containing different concentrations of ZnCl_2_.

## 2. Materials and Methods

### 2.1. Formulation of the Experimental Adhesive Systems

A model adhesive blend was formulated using the monomers HEMA, BISGMA, UDMA, and GDMA (Esstech Inc., Essington, PA, USA). Ethanol and water were used as solvents and Camphorquinone and EDMAB (ethyl N,N-dimethyl-4aminobenzoato) (Aldrich Chemical Company, Inc., Milwaukee, WI, USA) were incorporated as a photosensitizer and a reducing agent (Table 1). These chemicals were weighed in an analytical balance (AUW 220D, Shimadzu, Tokyo, Japan), then mixed, and homogenized in a dual centrifuge (150.1 FVZ SpeedMixer DAC, FlackTek, Inc., Hamm, Germany) at 2400 rpm for 2 min. Afterwards, ZnCl_2_ was added in concentrations (wt.%) of 0%, 2.0%, 3.5%, and 5.0% (Sigma-Aldrich, St. Louis, MO. #1208465) to the model adhesive, obtaining four different experimental adhesives (Zn0, Zn2, Zn3.5, Zn5, and SB). The adhesive system Adper Single Bond 2 (SB) was used as a commercial reference. The compositions of all adhesive systems are shown in Table 1.

All adhesives were light cured using a quartz-tungsten-halogen unit (Optilux 501 Demetron Inc., Danbury, CT, USA), at an irradiance of 650 mW/cm^2^, which was monitored using a radiometer (model 100, Demetron Inc., Danbury, CT, USA). This study followed the methods of da Silva et al., 2015 [17].

### 2.2. Degree of Conversion (DC)

One drop (0.785 mm^3^) of each adhesive system (*n* = 6) was inserted into a Teflon mold, positioned onto an ATR crystal of the FT-IR spectrometer (Alpha-P/Platinum ATR Module, Bruker Optics GmbH, Ettlingen, Germany) and the spectra between 1500 and 1800 cm^−1^ were recorded, using 40 scans at a resolution of 4 cm^−1^. Afterwards, the adhesive was light cured for 20 s and the spectra were recorded exactly as performed for the unpolymerized increments. The DC% was calculated from the ratio between the integrated area of absorption bands of the aliphatic C=C bond (1638 cm^−1^) to that of the C=O bond (1609 cm^−1^), used as an internal standard, which were obtained from the polymerized and unpolymerized increments, using the following equation:

(1) $$\begin{array}{c} DC\%=100\times \left[\;1-\left(\;\frac{R_{\text{polymerized}}}{R_{\text{unpolymerized}}}\;\right)\;\right], \end{array}$$

where *R* is integrated area at 1638 cm^−1^/integrated area at 1609 cm^−1^.

### 2.3. Water Sorption (WS) and Solubility (SO)

Firstly, the solvent from all adhesives was allowed to evaporate in air. Each adhesive (Table 1) was dispensed into a container (5.0 cm in diameter and 1.0 cm in depth) on an analytical balance with a precision of 0.01 mg (AUW 220D, Shimadzu, Tokyo, Japan), which was protected from ambient light to prevent premature polymerization. The initial mass was recorded and the specimens remained on the analytical balance until reaching mass equilibrium.

After solvent evaporation, disk-shaped specimens with 1 mm in thickness and 6 mm in diameter were prepared with a metallic mold for each adhesive (*n* = 6). A micropipette was used to dispense the adhesives directly into the mold. All visible air bubbles were carefully removed using a hypodermic needle. After, a polyester strip and glass slide were placed on top of the mold and the adhesives were light cured for 20 s. The disks were removed from the mold and light cured from the bottom surfaces for 20 s. The top and bottom surfaces of all disks were manually polished using a 4000-grit SiC paper (Arotec, Cotia, SP, Brazil) to eliminate any surface irregularities.

Water sorption and solubility tests were based on the ISO 4049 Standard (2000), except for the size of the specimen and for the period of immersion, which followed other studies [17, 29]. Initially, the disks were stored in a desiccator containing dehydrated silica gel at 37°C and weighed daily using an analytical balance with a precision of 0.01 mg (AUW 220D, Shimadzu, Tokyo, Japan) until a constant mass was attained (*m*_1_), during three consecutive days.

The thickness and diameter of each disk were measured at four points using a digital caliper (MPI/E-101, Mitutoyo, Tokyo, Japan), and the volume (*V*) was calculated in mm^3^. The specimens were then individually placed in sealed glass vials containing 10 mL of distillate water at 37°C. The immersion media were renewed every 7 days to prevent the proliferation of fungi and bacteria. The disks were gently wiped with soft absorbent paper and weighed at 24 hours intervals until a constant mass was attained (*m*_2_), during three consecutive days. Afterwards, the specimens were again put in a desiccator containing dehydrated silica gel at 37°C again and weighed daily until a constant mass was obtained (*m*_3_). Sorption (WS) and solubility (SO) in *μ*g/mm^3^ were calculated using the following formulas:

(2) WS=m2−m3V,SO=m1−m3V.

### 2.4. Microtensile Bond Strength (*μ*TBS)

Fifty caries-free extracted human third molars (collected after the patient's informed consent and approval from the Ethical Committee, Faculty of Medicine/UFF-CEP CMM/HUAP, protocol # 925.236), were disinfected in 0.5% chloramine T solution for one week and stored in distilled water until their use, which occurred within six months after extraction. A flat dentin surface was exposed after wet grinding the occlusal enamel of teeth using a 180-grit silicon carbide paper, followed by a 600-grit silicon carbide paper for 60 seconds to standardize the smear layer. Subsequently, teeth were divided into the five groups according to the adhesive systems (Table 1, *n* = 10). After dentin hybridization as described in Table 1, composite blocks were built up using two increments of 2 mm thick (Filtek Z250 XT, 3 M ESPE, Seefeld, Germany), individually light cured for 40 s.

After storage in distilled water at 37°C for 24 h, the teeth were longitudinally sectioned in both the mesiodistal and buccal-lingual directions, across the bonded interfaces (Isomet 1000 precision saw, Buëhler, Lake Bluff, IL, USA) producing beams with a cross-sectional area of approximately 1 mm^2^. The bonded beams originated from the same tooth were randomly divided and assigned to be tested after 24 hours (I) or 12 months (Y) of storage in distilled water at 37°C. The distilled water was changed monthly and its pH was monitored every week.

After each period of storage, two beams of each experimental condition were preserved for the nanoleakage test. The remaining beams had their cross-sectional area measured with a digital caliper (MPI/E-101, Mitutoyo, Tokyo, Japan) and were individually fixed to a microtensile device (ODMT03d, Odeme Biotechnology, Joaçaba, SC, Brazil) using cyanoacrylate glue (Super Bonder Flex Gel Loctite, Henkel, São Paulo, SP, Brazil) and subjected to a tensile load at a crosshead speed of 1.0 mm/min until failure (Emic DL2000, Instron Brazil, São José dos Pinhais, PR, Brazil). The *μ*TBS (MPa) was obtained by dividing the maximum load (N) by the cross-sectional area (mm^2^) of each tested beam. The fractured surfaces were evaluated under stereomicroscope at 40x magnification (SZ40, Olympus, Tokyo, Japan), and the mode of failure was classified as follows: adhesive (failures at the adhesive interface), cohesive (failures occurring in dentin or in resin composite), or mixed (mixture of adhesive and cohesive failure within the same fractured surface).

### 2.5. Nanoleakage (N)

For nanoleakage, the beams received two layers of nail varnish up to 1 mm from the bonding interface on both sides. Then, they were individually immersed in an aqueous ammoniacal silver nitrate solution (50% in weight; pH = 7.0) and kept in a dark environment for 24 h, thoroughly rinsed in running water, and immersed in a photo-developing solution (Kodak, Rochester, Nova York, USA) under fluorescent light for 8 h, in order to reduce silver ions into metallic silver grains at the bonding interface. Afterwards, the surfaces were wet polished with 600-grit, 1200-grit, and 4000-grit SiC paper, ultrasonically cleaned in water for 10 min (Ultrassom 750 USC, Quimis, Rio de Janeiro, Brazil), and dried for 48 hours in a desiccator with blue silica gel at 37°C.

The resin/dentin interfaces were observed using scanning electron microscopy (SEM) (Phenom ProX, Phenom-World, Eindhoven, Netherlands), at an accelerating voltage of 15 kV, backscattered mode, and using a charge reduction sample holder (low vacuum environment). Three images were registered for each beam: two from both ends (right and left sides) and one central, with a magnification of 2000x. The amount of silver nitrate uptake in the hybrid layer was registered as a percentage of the total area observed, using an Energy-dispersive X-ray spectroscopy (EDS) detector (Phenom ProX, Phenom-World, Eindhoven, Netherland).

### 2.6. Statistical Analysis

Data were analyzed using Statgraphics 5.1 software (Manugistics, Rockville, MD, USA). Statistic was performed at a significance level of *α* = 0.05. Firstly, the normal distribution of errors and the homogeneity of variances of the data were checked using Shapiro-Wilk's and Levene's tests, respectively. Based on these preliminary statistics, the *μ*TBS and nanoleakage data were analyzed using two-way ANOVA and Tukey's HSD post hoc test. DC%, WS, and SO data were analyzed using one-way ANOVA and Tukey's HSD post hoc test.

## 3. Results

The results of DC%, WS, and SO are depicted in Table 2. The highest DC% was presented by the commercial adhesive system SB, whereas Zn5 presented the lowest DC% (*p* < 0.05). No statistically significant difference was found for DC% among Zn0, Zn2, and Zn3.5 (*p* > 0.05). Regarding WS, the highest value was presented by Zn5 (*p* < 0.05), followed by Zn3.5. SB, Zn0, and Zn2 showed no differences in WS from each other (*p* > 0.05). With respect to SO, the highest value was presented by Zn5 (*p* < 0.05), followed by SB and Zn3.5 (*p* < 0.05). Zn0 and Zn2 presented the lowest values of SO (*p* > 0.05).

**Figure 1:**
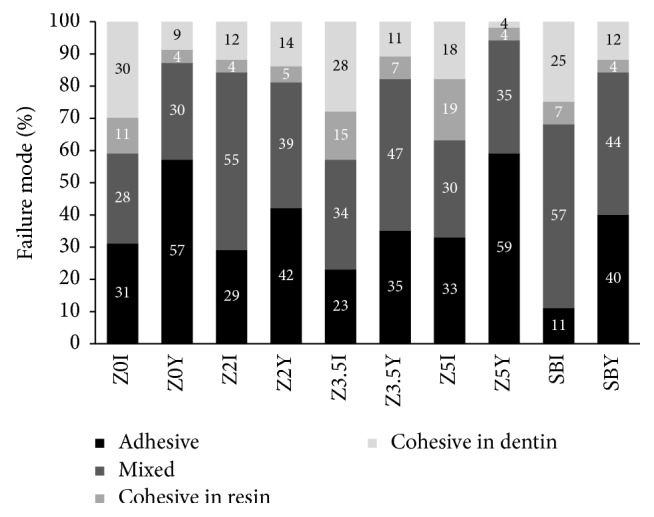
Failure mode after each period of storage in distilled water.

The results of *μ*TBS are shown in Table 3. No premature failure was observed during the *μ*TBS experiment. Only the experimental adhesive systems containing ZnCl_2_ (Zn2, Zn3.5, and Zn5) were capable of maintaining the resin-dentin bonding stability after 12 months of water storage (*p* < 0.05). The highest *μ*TBS was presented by SB after 24 h of water storage (54.15 MPa, *p* < 0.05). However, this commercial adhesive system presented the greatest reduction in *μ*TBS (30.34 MPa) after 12 months of water storage (43.97%, *p* < 0.05).  Figure 1 shows the result of the failure mode evaluation. Except Zn2, all the other adhesive systems showed a predominance of adhesive failures after 12 months of water storage, with this change being more remarkable for SB.

**Figure 2:**
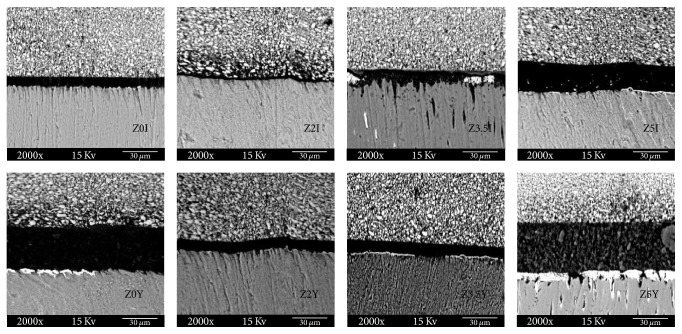
Representative backscattered SEM micrographs of the resin-dentine adhesive interfaces for the experimental groups.

With respect to nanoleakage (Table 2), only Zn2 and Zn3.5 did not suffer an increase on this phenomenon after 12 months of water storage (*p* < 0.05). Zn0 suffered the greatest nanoleakage in both times of evaluation (*p* < 0.05).  Figure 2 shows representative SEM images of nanoleakage for all experimental adhesive systems. It can be noted that the specimens of Zn0 and Zn5 presented a remarkable nanoleakage expression after 12 months of water storage.

## 4. Discussion

One of the targets of the dental scientists is to develop restorative biomaterials able to survive the harsh conditions found in the oral environment. In the field of adhesive dentistry, the perfect picture of this is to create adhesive systems resistant to hydrolytic degradation and capable of maintaining the stability of collagen fibrils exposed by acidic substances, that is, phosphoric acid and phosphate acidic monomers, applied during the adhesive protocol. In other words, create stable adhesive structures over time. Different from former studies in which Zn salts were added to commercially available dental adhesives [23–25], in the current study different concentrations of ZnCl_2_ were incorporated into a model adhesive blend, which was formulated based on the composition of Single Bond 2, as described by its manufacturer. This was done to analyze the real effects of ZnCl_2_, avoiding the influence of unknown ingredients present in commercially available adhesive systems, as well as their contents, on the obtained results. Also, Single Bond 2 was used as a commercial control to verify if the performance of the experimental adhesives tested here would be in agreement with the properties of a commercial, well-proven, adhesive system widely used in the clinical practice.

Although the ZnO has been deeply investigated [20, 21, 23–25, 28], ZnCl_2_ was chosen to this study and added to model adhesive blend thanks to its higher solubility in water and ethanol than ZnO and to its fast rate of dissolution in Zn^2+^ [20], which would make easier its incorporation into the adhesive. Also, in a previous pilot study carried out in this lab (unpublished data), it was observed that when added to the model adhesive this Zn salt produced less opaque solutions than ZnO blends, an aspect crucial to the polymerization behavior of the experimental adhesives [21, 22].

A high degree of monomer conversion is the first step to an adhesive system that develops suitable physicochemical properties and performs well when applied into the oral cavity [30, 31]. In the current study, the degree of conversion ranged from 64.41% to 73.50%, values that agree with those presented by commercially available adhesive systems [32, 33], and with other experimental adhesives containing different Zn salts [22]. On the other hand, Zn5 presented a significantly lower degree of conversion than the other model adhesive blends, leading to the acceptation of the first hypothesis.

It is well known that the match between the refractive index of the filler particles and the organic matrix is mandatory to a polymeric restorative biomaterial reaching suitable translucency [34] and that this strongly influences its light-polymerization behavior. Thus, taking into account the fact that methacrylate organic matrixes have a refractive index close to 1.5 [35] and that of ZnCl_2_ is approximately 1.681 [36], it is reasonable to infer that the concentration of 5 wt.% of ZnCl_2_ could have increased the mismatching between the refractive index of both organic matrix and ZnCl_2_, thereby increasing the Zn5 opacity. On the other hand, it is plausible to claim that the concentrations of 2 and 3.5 wt.% of ZnCl_2_ did not interfere with the translucency of Zn2 and Zn3.5 adhesive blends.

Indeed, Barcellos et al. [21] showed that the incorporation of 2, 5, and 10 wt.% of ZnO impaired the degree of conversion of model dentin adhesives, suggesting that this was due to a decrease in light penetration through the adhesives because of the high opacity of ZnO. According to these authors, this behavior could decrease the crosslink density and form structural defects in the cross-linked adhesive polymer, aspects highly deleterious to the clinical performance of adhesive systems. Thus, considering that the refractive index of ZnCl_2_ (1.681) [36] is closer to that of the organic matrix (1.5) than that of ZnO (2.004) [37], it is reasonable to defend that ZnCl_2_ could be more suitable than ZnO for developing bioactive adhesive systems composed of methacrylate monomers like those used in the present model blend.

The transport of oral fluids (saliva, organic acids produced by the oral biofilm and water) through the adhesive polymer structure is crucial to the degradation of the tooth-adhesive interfaces [3, 38, 39], and this has been related to the impairment of some physical properties of the adhesive systems [40]. Thus, the evaluation of water sorption and solubility is crucial to analyze the performance of innovative-bioactive adhesive systems [17, 41]. In the current study, the experimental adhesive system with 2 wt.% of ZnCl_2_ did not suffer higher water sorption and solubility than the model blend Zn0 (control). This result disagrees with the study of Pomacondor-Hernandez et al. [28], where the commercially available adhesives Adper Single Bond Plus (3M-ESPE, St Paul, MN, USA) and Clearfil SE Bond (Kuraray, Tokyo, Japan) modified with 2 wt.% of ZnCl_2_ presented higher water sorption and solubility than their unmodified counterparts. This disagreement between those results and the current ones could be related to the differences of methodologies employed in both studies. The authors of that study did not allow the adhesive solvents (ethanol and water) to perform in the same way as in this study, where they were allowed to evaporate before the WS and SO specimens building. Thus, it is reasonable to infer that the evaporation of solvents from the specimens during drying (*m*_1_) could have increased the values of solubility and that the voids produced after the solvent evaporation could have also allowed a greater sorption of water during the storage period (*m*_2_). The comparison between the values of WS and SO of Single Bond 2 obtained here (137.0 mm/mm^3^ and 23.37 *μ*/mm^3^, resp.) and in the study of Pomacondor-Hernandez et al. [28] (256.5 mm/mm^3^ and 84.5 *μ*/mm^3^, resp.) may reinforce this thought.

On the other hand, the sorption and the solubility of Zn3.5 and Zn5 were significantly higher than the control group (Zn0), with the values of Zn5 (WS = 285.44 *μ*g/mm^3^ and SO = 30.21 *μ*g/mm^3^) being, by rounding off, twofold compared to those of the model blend Zn0 (WS = 130.26 *μ*g/mm^3^ and SO = 16.51 *μ*g/mm^3^). These results can be explained by the high solubility of ZnCl_2_ in water (432 g/100 ml at 25°C) [20], which could have been leaching out with the water during the drying of the specimens, consequently creating a high level of porosities that favored the accommodation of greater amounts of water within the adhesive polymer networks. These aspects also support the acceptation of the first hypothesis of the present study.

It is important to emphasize that although some degradation of the adhesive from the hybrid layer could occur during long-term water storage [40, 42], in the current study this was not high enough to mask the positive effects of the incorporation of the 2 wt.% of ZnCl_2_ into model adhesive blends, on the resin-dentin bonding stability.

The current results showed that the addition of ZnCl_2_ to the adhesive systems was capable of maintaining the resin-dentin bonding stability after 1 year of water storage. Moreover, it was also proved that Zn2 and Zn3.5 did not suffer a significant increase in nanoleakage expression after this period. Thus, the second hypothesis of the present study was also accepted. To the best of the authors' knowledge, these are the longest results regarding the efficacy of Zn salts added to adhesive systems on these responses. Specifically regarding resin-dentin bonding stability, the present results support those of Toledano et al. [43] and Barcellos et al. [21], who showed that ZnO-Single Bond and a ZnO-model adhesive maintained the resin-dentin bond stability after 3 and 6 months of water storage, respectively. Osorio et al. [44] showed a reduction in collagen degradation mediated by MMP-2 in phosphoric acid demineralized dentin of 24% and 60% at 24 h and 3-week period, respectively, when ZnCl_2_ was added to the media (artificial saliva). Furthermore, Osorio et al. [45] also showed that the hybridization of demineralized dentin with ZnCl_2_-Single Bond reduced the collagen degradation by 30.2% in comparison with unmodified Single Bond. These studies clearly confirm the MMP inhibition potential of Zn and can be used to explain the resin-dentin bond stability presented by experimental etch-and-rinse adhesives containing ZnCl_2_ in the present study.

On the other hand, even though the experimental adhesives Zn3.5 and Zn5 have maintained the resin-dentin bond stability after 1 year of water storage, their values of *μ*TBS presented a reduction of 17.9% and 23%, respectively, at 24 h and of 14.0% and 23.7%, respectively, after 1 year when compared to the control group Zn0. Most probably, this can be related to the significant higher WS and SO presented by them both and to the lower DC% presented by Zn5. It has been shown that a high water sorption and solubility [17, 41, 42] and a low degree of conversion [32] can jeopardize the polymeric adhesive structures and negatively influence the resin-dentin bond and the nanoleakage expression.

From Figure 1, it can be noted that the adhesive Zn2 was the only one that presented a similar percentage of adhesive failures at both periods of evaluation. This can be taken as the adhesive interface formed by Zn2 being more stable than the others and reinforces that 2 wt.% of ZnCl_2_ could be more suitable to formulating bioactive etch-and-rinse adhesive systems.

Beside its effect as MMP inhibitor [44], previous studies have advocated that Zn salts may stimulate the biomineralization and remineralization of the hard dental tissues [23, 46, 47]. Most probably, both mechanisms had influenced the stability of the nanoleakage expression presented by Zn2 and Zn3.5 in the current study. It is possible that, after phosphoric acid etching, the presence of the remaining Ca^2+^ within the collagen scaffold could have favored the remineralization of apatite crystals around the exposed collagen fibrils by Zn^2+^, thereby interfering with MMP activity and reinforcing the hybrid layer [25]. Moreover, the faster release of Zn^2+^ may have also stabilized the exposed collagen fibrils against enzymatic degradation through crosslinking, consequently improving the collagen fibrils remineralization [47].

In the current study, Single Bond 2 was used as a commercial reference to infer whether the performance of the adhesive systems containing ZnCl_2_ would be in agreement with that presented by a commercially, well-proven, adhesive system. Except for the *μ*TBS at 24 h, all the other values obtained by the adhesives containing ZnCl_2_ were close to those presented by Single Bond 2. Also, irrespective of the highest *μ*TBS at 24 h, Single Bond 2 was not capable of maintaining the resin-dentin bonding stability after 1 year of water storage. The greater increase in the number of adhesive failures after this period (Figure 1) can be seen as a clear signal of the deterioration of the adhesive interfaces produced by SB over time. Based on these aspects, it is reasonable to claim that the incorporation of ZnCl_2_ into etch-and-rinse adhesive systems may be seen as a suitable strategy to improve the adhesive interface over time.

## 5. Conclusions

It can be concluded that the addition of 2 wt.% of ZnCl_2_ avoided not only the drop of the resin-dentin bonding strength but also the increase of the nanoleakage after one year of water storage, without compromising the physicochemical properties of the etch-and-rinse adhesive systems evaluated. Although this procedure had shown good results in the current study, as ZnCl_2_ is a high soluble salt, a high lixiviation in the oral environment could also occur, affecting its action over time. So, further investigations extending the time of water immersion, as well as clinical studies, are needed. Besides, it must be investigated if other physical properties would be compromised with this addition.

## Figures and Tables

**Table 1:** Composition and mode of application of the adhesives.

Adhesives	ZnCl_2_ (wt.%)	Composition (wt.%)	Mode of application
Zn0 (control)	0	BisGMA (20%), HEMA (20%), UDMA (10%), ethanol (30%), water (4%), GDMA (15%), Camphorquinone (0.5%), EDMAB (0.5%)	Two consecutive layers were applied by means of a microbrush with an active mode on the dentin. The surface was gently air-dried and light cured for 20 s
Zn2	2		
Zn3.5	3.5		
Zn5	5		
SB	0	BisGMA (10–20%), HEMA (5–15%), ethanol (25–30%), water (<5%), GDMA (5–10%), UDMA (1–5%) copolymer of acrylic and itaconic acids, silane treated silica (nanofilled–10–20%) diphenyliodonium hexafluorophosphate (<0.5%), EDMAB (<0.5%)	

HEMA: 2-hydroxyethyl methacrylate; GDMA: glycerol 1,3-dimethacrylate; BisGMA: bisphenol A diglycidyl ether dimethacrylate; UDMA: diurethane dimethacrylate; EDMAB: ethyl 4-dimethyl aminobenzoate.

**Table 2:** Means and standard deviations of DC (%), WS (*µ*g/mm^3^), and SO (*µ*g/mm^3^) for each adhesive.

Adhesives	DC%	WS	SO
Zn0 (control)	68.67 (1.63)^b^	130.26 (3.86)^a^	16.51 (0.41)^a^
Zn2	67.46 (0.68)^b^	139.76 (1.39)^a^	16.87 (0.64)^a^
Zn3.5	66.89 (0.92)^b^	180.70 (3.04)^b^	18.57 (0.68)^b^
Zn5	64.41 (0.63)^a^	285.44 (9.28)^c^	30.21 (2.10)^d^
SB	73.50 (1.15)^c^	137.00 (8.88)^a^	23.37 (2.54)^c^

In columns, different letters indicate statistical differences (Tukey's HSD test, *α* = 0.05).

**Table 3:** Means and standard deviations of *µ*TBS (MPa) and nanoleakage (%) after each storage time in distilled water.

Adhesives	*µ*TBS (MPa)		Nanoleakage (%)	
	24 hours (I)	12 months (Y)	24 hours (I)	12 months (Y)
Zn0 (control)	34.62 (2.50)^Aa^	31.53 (2.42)^Ab^	0.51 (0.07)^B,a^	1.26 (0.25)^C,b^
Zn2	32.31 (2.05)^Aa^	31.41 (2.06)^Aa^	0.23 (0.07)^A,a^	0.33 (0.07)^A,a^
Zn3.5	28.44 (0.57)^Ba^	27.13 (0.66)^Ba^	0.29 (0.09)^A,B,a^	0.41 (0.09)^A,a^
Zn5	26.66 (0.76)^Ba^	24.06 (1.10)^Ba^	0.37 (0.09)^A,B,a^	0.94 (0.20)^B,b^
SB	54.15 (2.33)^Ca^	30.34 (5.42)^Ab^	0.22 (0.04)^A,a^	0.51 (0.06)^A,b^

In rows, for each property, different lowercase letters mean statistical difference (Tukey's HSD test, *α* = 0.05). In columns, different uppercase letters mean statistical difference (Tukey's HSD test, *α* = 0.05).
